# Impact of tricuspid regurgitation and right ventricular dysfunction on outcomes after transcatheter aortic valve replacement: A systematic review and meta‐analysis

**DOI:** 10.1002/clc.23126

**Published:** 2018-12-22

**Authors:** Jiaqi Fan, Xianbao Liu, Lei Yu, Yinghao Sun, Sanjay Jaiswal, Qifeng Zhu, Han Chen, Yuxin He, Lihan Wang, Kaida Ren, Jian'an Wang

**Affiliations:** ^1^ Zhejiang University School of Medicine Hangzhou People's Republic of China; ^2^ Department of Cardiology Second Affiliated Hospital Zhejiang University School of Medicine Hangzhou People's Republic of China; ^3^ Department of Echocardiography, The Second Affiliated Hospital Zhejiang University School of Medicine Hangzhou China; ^4^ Department of Cardiology Guangdong People's Hospital Guangzhou China

**Keywords:** all‐cause mortality, prognosis, right ventricular function, transcatheter aortic valve replacement, tricuspid regurgitation

## Abstract

Far less attention has been paid to the prognostic effect of right‐side heart disease on outcomes after transcatheter aortic valve replacement (TAVR) when compared with the left side. Therefore, we performed a systematic review and meta‐analysis on the impact of tricuspid regurgitation (TR) and right ventricular (RV) dysfunction on outcomes after TAVR. We hypothesized that TR and RV dysfunction may have a deleterious effect on outcomes after TAVR. Article revealing the prognostic effect of TR and RV dysfunction on outcomes after TAVR were being integrated. Random or fixed effect model was adopted in accordance with the heterogeneity. There were nine studies with a total of 6466 patients enrolled after a comprehensive literature search of the MEDLINE/PubMed, EMBASE, ISI Web of Science, and Cochrane databases. The overall analysis revealed that moderate or severe TR at baseline increased all‐cause mortality after TAVR (HR = 1.79, CI 95% 1.52‐2.11, *P* < 0.001). Both baseline RV dysfunction (HR = 1.53, CI 95% 1.27‐1.83, *P* < 0.001) and presence of RV dilation (HR = 1.83, CI 95% 1.47‐2.27, *P* < 0.001) were associated with all‐cause mortality. Both baseline moderate or severe TR and RV dysfunction worsen prognosis after TAVR and careful assessment of right heart function should be done for clinical decision by the heart team before the TAVR procedure.

## 1 INTRODUCTION

Transcatheter aortic valve replacement (TAVR) is a novel alternative to inoperable, high risk even moderate risk symptomatic severe aortic stenosis (AS) patients. However, short‐ and long‐term morbidity and mortality after TAVR are still an issue of concern.^1^, ^2^ Several predictors of outcome after TAVR are well established,^3^, ^4^ such as moderate or severe aortic regurgitation, new‐onset left bundle branch block, pulmonary artery hypertension, reduced left ventricular ejection fraction (LVEF).

Recently, more and more studies are paying attention to the prognosis of tricuspid regurgitation (TR) and right ventricular (RV) dysfunction on outcome after aortic valve replacement.^5^, ^6^, ^7^, ^8^, ^9^, ^10^, ^11^, ^12^, ^13^, ^14^, ^15^ In surgical valve aortic replacement, RV function is an independent predictor of all‐cause mortality after the procedure, and whether TR can be regarded as an independent predictor is still controversial.^16^, ^17^, ^18^ However, in TAVR, the prognosis of RV function and TR on outcomes is contradictory. A study in the subgroup of PARTNER shows that moderate or severe TR and RV sizes are associated with increased all‐cause mortality, but RV dysfunction is not.^6^ While in a recent‐single center prospective registry study, only RV function, but not TR remained associated with outcome after TAVR.^8^ Therefore, we performed a systematic review and meta‐analysis of the literature to assess the impact of baseline TR and RV dysfunction on outcome after TAVR.

## 2 METHODS

### 2.1 Search strategy

We searched the MEDLINE/PubMed, EMBASE, ISI Web of Science and Cochrane databases for studies without region and language restrictions from the earliest date possible up to 28 February 2017. The term searched were ([TAVR] OR [TAVR] OR [transcatheter valve] OR [transcatheter aortic valve] OR [transcatheter heart valve] OR [percutaneous valve] OR [percutaneous aortic valve]) AND ([right ventricular dysfunction] OR [tricuspid regurgitation]) AND ((outcome) OR (survival) OR (prognosis) OR (predictor)). When data were considered to have an overlap, only the most recent paper was included. A systematic review was conducted in accordance with the preferred reporting items for systematic reviews and meta‐analyses (PRISMA) and meta‐analysis of observational studies in epidemiology (MOOSE) guidelines.^19^, ^20^

### 2.2 Selection criteria

Studies were included if they met the following criteria: (a) Reported data on the association between RV dysfunction or TR severity and outcomes after TAVR expressed as hazard ratio (HR), (b) Reported to have enrolled at least 100 patients. Exclusion criteria were: (a) No clear definition on RV dysfunction and RV size; (b) No clear statement on follow‐up duration; (c) Patients received medical therapy or surgical aortic valve replacement; (d) Abstract, case report, conference presentations, reviews, or editorials.

### 2.3 Data extraction, endpoints, and definition

Two reviewers independently screened the articles for eligibility according to the inclusion and exclusion criteria. The reviewers compared the selected studies and any discrepancy was resolved by consensus with a third reviewer.

TR severity was graded in these works of literature as none/trace (grade 0), mild (grade 1), moderate (grade 2), or severe (grade 3) integrating structural, Doppler, and quantitative parameters according to the American Society of Echocardiography, including assessment of vena contracta width, proximal isovelocity surface area radius, tricuspid valve morphology, right atrial (RA) and RV size, inferior vena cava size, jet area, jet density and contour, and hepatic vein flow.^21^ Moderate and severe TR were categorized as “significant TR” while none, trace and mild as “nonsignificant TR.”

According to ASE guideline,^22^ RV function includes RV systolic function (at least one of the following: fractional area change [FAC], tissue Doppler‐derived tricuspid lateral annular systolic velocity [S′], and tricuspid annular plane systolic excursion [TAPSE]; with or without RV index of myocardial performance [RIMP]; and RV ejection fraction) and RV diastolic function (the early [E‐wave] and late diastolic [A‐wave] tricuspid velocities [E/A ratio], deceleration time, the medial and lateral peak early diastolic velocity [E′] [E/E′ ratio], and RA size). RV size is assessed by longitudinal diameter, basal and mid diameter at the end of diastole in right ventricle‐focused apical four‐chamber view.

Information extracted included author(s), publication year, study region(s) and design, included patients number, type of device and approach, duration of follow‐up, baseline characteristics of patients, and outcomes of interest. We extracted hazard ratios (HRs) with their corresponding 95% confidence intervals (CIs) from the included studies.^23^ The primary endpoint was all‐cause mortality.

### 2.4 Data analysis and synthesis

Meta‐analysis was performed in RevMan Software Version 5.3 and Stata Software Version 14.0. Heterogeneity was assessed by *I* ^*2*^ index, with 25%, 50%, and 75% representing low, moderate, and high heterogeneity, respectively. When the heterogeneity of meta‐analysis was ≥50%, we adopted the random effects model, and when the heterogeneity was <50%, we used the fixed effect model. Given the number of the included studies was less than 10, publication bias was not assessed.^24^ A *P* < 0.05 (two‐tailed) was considered significant. We also performed the meta‐analysis to figure out the impact of TAPSE, FAC, RIMP, and S′ on all‐cause mortality after TAVR. Sensitivity analysis was performed by removing one study at a time to test the robustness of the results. The quality of the enrolled studies was evaluated by two independent reviewers according to the Newcastle‐Ottawa Scale ranging from 0 to 8.

## 3 RESULTS

### 3.1 Search strategy, population characteristics, and descriptions

**Figure 1:**
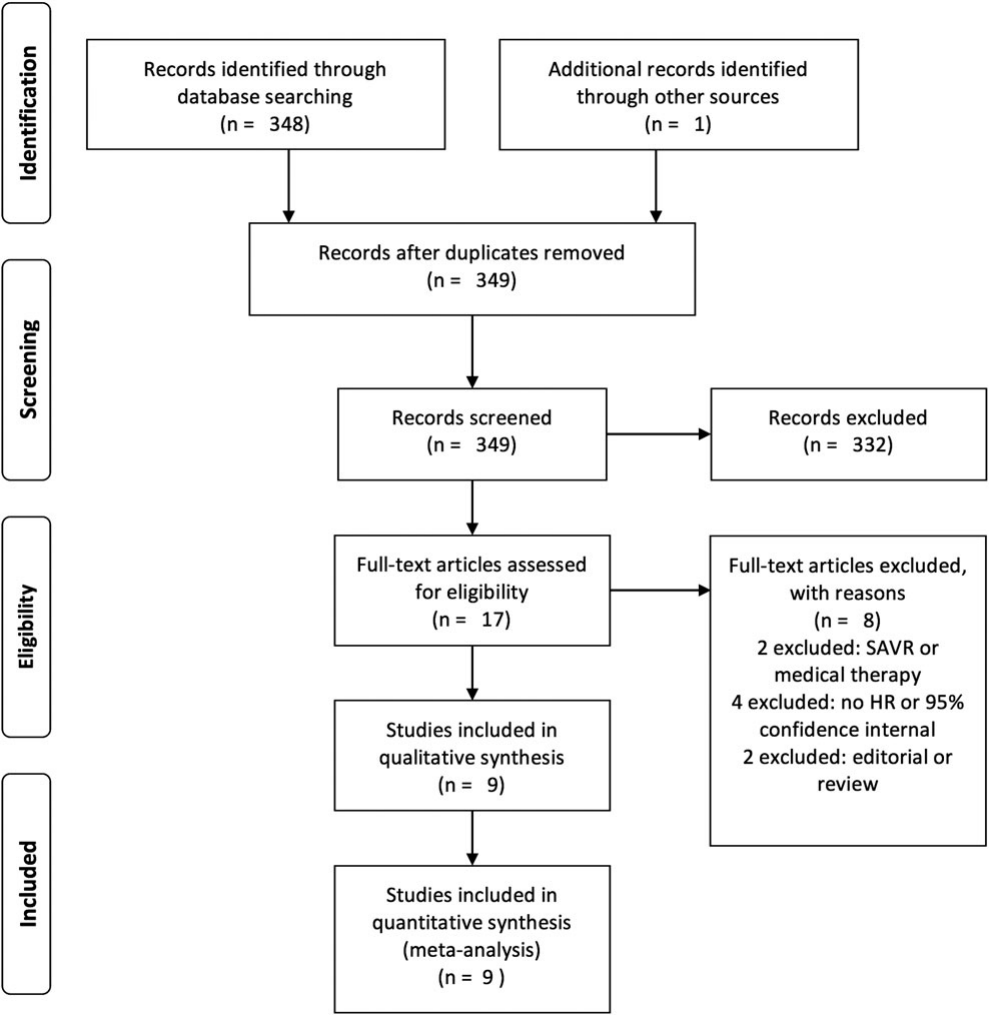
PRISMA flow diagram demonstrating study selection

A total of 349 records were analyzed: 348 identified through database searching and one through references (Figure 1). After the first evaluation of titles and abstracts, 349 records were screened and 332 of these were excluded. Seventeen studies were analyzed as full‐article (Figure 1). After excluded eight studies with reasons, we included nine studies with a total of 6466 patients (Figure 1). The characteristics of the enrolled studies and the quality ratings were listed in Table 1.

**Table 1:** Study characteristic and quality

Author	Year	Study design	Region	Number	Valve	Follow‐up	Study quality
Lindman 6	2015	Prospective	United States	542	ES/ESX	1 year	7/8
Schwartz 7	2016	Prospective	United States	519	NA	5 years	7.5/8
Ito 8	2016	Prospective	United States	282	ES	412 days	7/8
Barbanti 9	2015	Prospective	Canada	518	ES/ESX/ES3/central/MC/portico	2 years	7/8
Lindsay 10	2016	Prospective	United Kingdom	190	MC/ES	850 days	8/8
Poliacikova 11	2013	Prospective	United Kingdom	155	MC/ES	628 days	6/8
Schymik^12^	2015	Prospective	17 countries	2688	ESX	1 year	7.5/8
Testa^13^	2016	Prospective	Italy	870	MC	1 year	7/8
Griese^14^	2017	Prospective	Germany	702	ES/ESX	4 years	7/8

Abbreviations: ES, Edwards SAPIEN; ESX, Edwards SAPIEN XT; MC, Medtronic CoreValve.

The mean age was 82.17(±6.73) and 46% of patients were male (Table 2). Hypertension was present in 83% of the population, diabetes in 33%, significant mitral regurgitation (MR) in 23%, coronary artery disease in 51%, peripheral artery disease in 24%, and atrial fibrillation (AF) in 28% (Table 2). Mean pre‐procedure LVEF was 54% (±13%), pulmonary artery systolic pressure (PASP) was 44(±15) mmHg, EuroSCORE was 21(±13) and STS was 8(±6). NYHA III/IV was present in 80% of the population (Table 2).

**Table 2:** Baseline characteristics of the patients included in meta‐analysis

Author	Patient age, y	Male sex, %	Hypertension, %	Significant MR, %	DM, %	Cad, %	Pad,%	AF, %	PASP, mmHg	LVEF, %	Euro‐SCORE	STS	TF access, %	NYHA, III/IV, %
Lindman 6	84.6 ± 8.5	50.1	90.3	29.0	35.0	66.0	NA	36.9	41 (30‐58)	52.0 ± 12.6	NA	10.5 ± 5.5	NA	NA
Schwartz 7	85.6 ± 6.0	43.0	87.0	21.0	35.0	60.0	NA	16.4	42.5 ± 15.0	56.3 ± 9.0	20.5 ± 14	NA	NA	93.0
Ito 8	80.5 ± 7.9	55.6	88.1	9.7	40.3	61.2	61.2	23.9	45.4 ± 15.0	54.6 ± 12.9	NA	9.8 ± 5.1	NA	90.3
Barbanti 9	81.5 ± 8.4	55.1	77.6	40.1	30.1	NA	27.5	38.2	43.7 ± 17.8	53.9 ± 13.9	NA	8.3 ± 5.2	66.2	86.7
Lindsay 10	80.2 ± 5.3	50.0	NA	NA	74.7	32.6	17.4	20.0	35(33‐38)	62(59‐67)	NA	NA	68.9	73.2
Poliacikova 11	81.5 ± 6.2	49.0	NA	NA	25.0	61.9	NA	NA	NA	NA	9.6 ± 1.9	NA	87.1	NA
Schymik^12^	81.4 ± 6.6	42.3	80.9	19.8	29.4	44.2	21.2	25.6	44.9 ± 14.9	54.4 ± 12.5	20.4 ± 12.4	7.9 ± 6.6	62.7	76.9
Testa^13^	82.6 ± 5.1	47.4	NA	23.2	28.3	NA	20.2	15.4	NA	NA	23.0 ± 13.2	6.0 ± 1.5	91.6	72.1
Griese^14^	82.0 ± 5.0	42.0	NA	NA	36.0	58.0	25.0	50.0	NA	52.0 ± 14.0	21.0 ± 15.0	NA	49.1	83.0

Abbreviations: AF, atrial fibrillation; CAD, coronary artery disease; LVEF, left ventricular ejection fraction; DM, diabetes mellitus; MR, mitral regurgitation; NA, not available; NYHA, New York Heart Association; PAD, peripheral artery disease; PASP, pulmonary artery systolic pressure; STS, Society of Thoracic Surgery; TF, transfemoral.

The TR severity is divided into two comparable groups, none/trace/mild TR, and moderate/severe TR. The RV function is categorized by normal or abnormal. All descriptions of RV dysfunction and RV size in included studies were shown in Supporting information Table S1.

### 3.2 Outcomes

**Figure 2:**
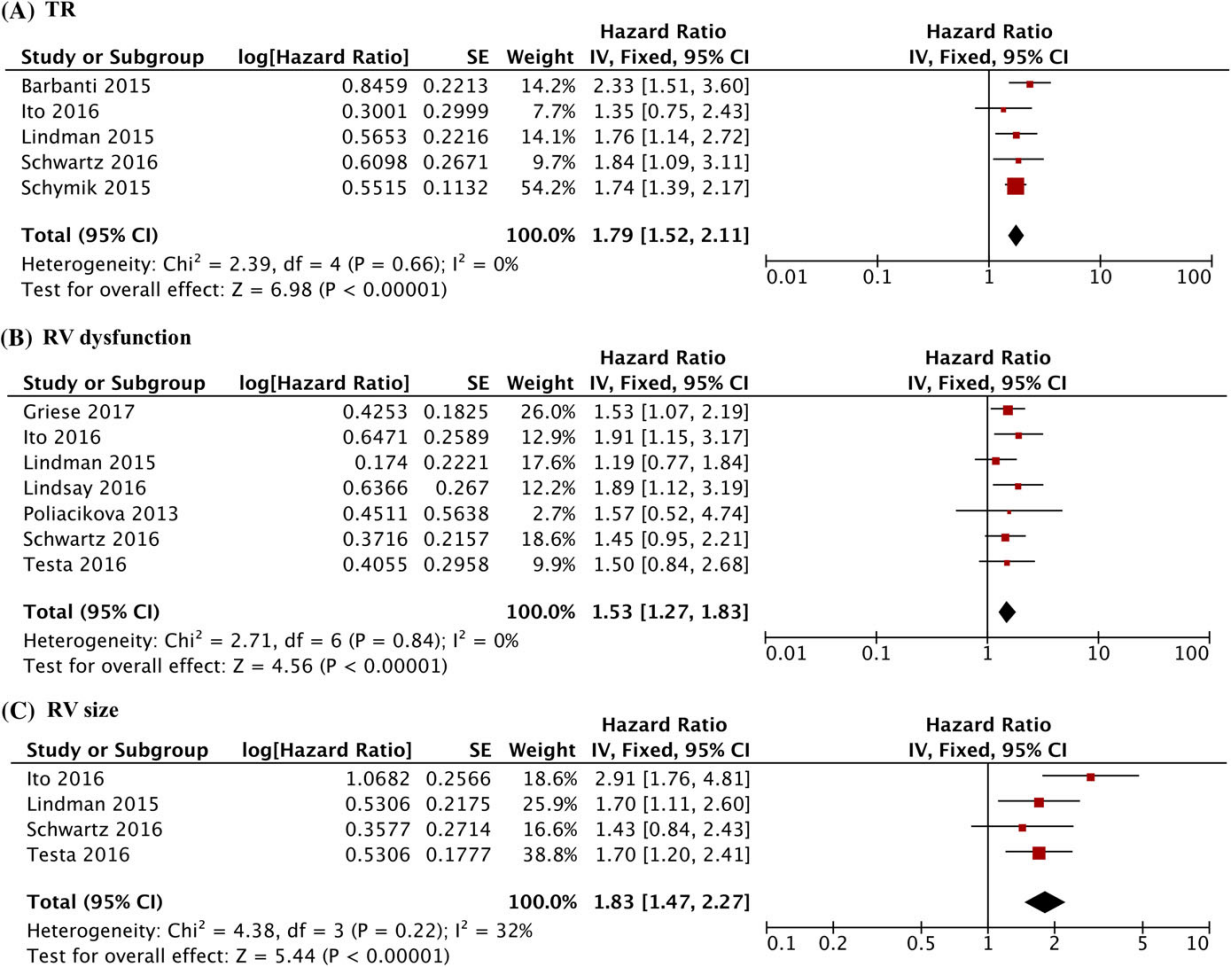
All‐cause mortality outcomes after TAVR. Forest plot showing the individual and pooled analysis for hazard ratio of (A) tricuspid regurgitation (B) right ventricular dysfunction (C) right ventricular dilation on all‐cause mortality

Patients with moderate or severe TR were associated with increased all‐cause mortality significantly (HR = 1.79, CI 95% 1.52‐2.11, *P* < 0. 00001) (Figure 2A) compared with no/trace or mild TR. Patients with RV dysfunction had higher all‐cause mortality (HR = 1.53, CI 95% 1.27‐1.83, *P* < 0.0001) (Figure 2B) compared with normal RV function. Preoperative RV dilatation increased the all‐cause mortality (HR = 1.83, CI 95% 1.47‐2.27, *P* < 0.00001) (Figure 2C).

**Figure 3:**
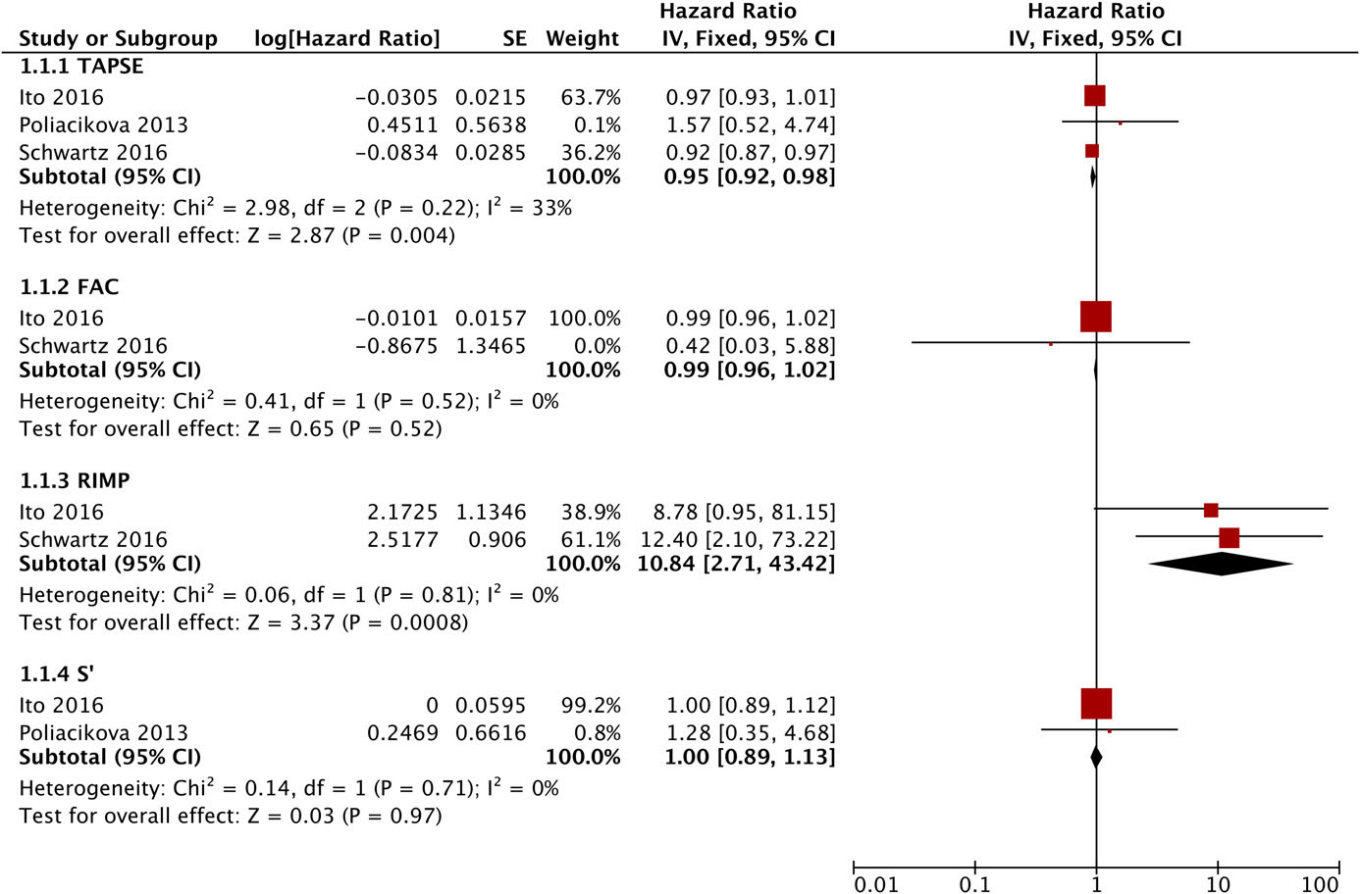
Forest plot demonstrating the individual and pooled analysis for hazard ratio of tricuspid annular plane systolic excursion (TAPSE), fractional area change (FAC), RV index of myocardial performance (RIMP) and tricuspid lateral annular systolic velocity (S′) on all‐cause mortality after TAVR

The results of meta‐analysis about patients with different methods of RV function assessment were shown in Figure 3. However, the results were different from the RV dysfunction. The TAPSE and the RIMP was associated with the increased all‐cause mortality (TAPSE: HR = 0.95, CI 95% 0.92‐0.98, *P* = 0.004; RIMP: HR = 10.84, CI 95% 2.71‐43.42, *P* = 0.0008) (Figure 3). But the impact of the FAC and S′ on all‐cause mortality was not significant (FAC: *P* = 0.52; S′: *P* = 0.97). The statistical heterogeneity was 33% in the group of TAPSE while other groups were 0 (Figure 3).

### 3.3 Sensitivity analysis

When the sensitivity analysis was performed by removing one study at a time, the overall effect of TR and RV dysfunction on all‐cause mortality were not changed. When it comes to RV size on all‐cause mortality, the situation was different. After removing the study of Saki Ito, the *I* ^*2*^ of the meta‐analysis decreased to 0, the *P* value of the heterogeneity to 0.85, while the overall effect of RV size and its significance remained unchanged (HR = 1.64, CI 95% 1.29‐2.09, *P* < 0.0001) (Figure S1).

## 4 DISCUSSION

This is actually the first meta‐analysis to evaluate the impact of right heart function on outcomes after TAVR. We included nine studies enrolling 6466 patients and found that (a) preoperative TR is assigned to increased all‐cause mortality after TAVR, (b) preoperative RV dysfunction is related to all‐cause mortality which is often related to the influence of TAPSE and RV myocardial performance, (c) pre‐TAVR RV size could possibly be linked to increased all‐cause mortality.

Although the significant TR late in left heart valve procedure is apparent,^16^ the prognostic impact of baseline significant TR continues to be a topic of debate, particularly in TAVR. The research by Lindman et al^6^ highlights the prognostic impact of TR on survival after TAVR, however, it is affected by the existence of moderate or severe MR. Other studies^8^, ^9^, ^10^ also show a lower rate of survival in moderate or severe TR group, however, the hazard ratio of moderate or severe TR is not substantial anymore following being adjusted by other echocardiographic and clinical variables, for example, LVEF, PASP, MR, AF. This reminds us that TR in those patients with severe AS is within an advanced disease stage. Other echocardiographic and clinical factors may play an even more important role in the prognosis after TAVR. In our meta‐analysis, we discover that not only the unadjusted TR but also the TR adjusted by clinical factors and echocardiographic factors is the prognostic effect of the all‐cause mortality after TAVR (Figure S2). However, more studies have to be carried out to verify this result.

Few studies provided data on the impact of the baseline RV dysfunction on outcome after SAVR or TAVR. Baseline RV dysfunction worsens the short‐term outcomes after surgical aortic valve replacement (SAVR).^5^, ^25^, ^26^ However, the prognostic impact of baseline RV dysfunction on outcomes after TAVR is not well established yet. In our pooled analysis, we found that the coexistence of baseline RV in patients with AS is associated with increased all‐cause mortality after TAVR. We advise heart team that RV function assessment should be more considered for TAVR and as a predictor of survival after TAVR based on our results in this meta‐analysis.

This negative effect could be attributed to the following pathophysiological mechanisms. TR is considered to be caused by dilation of the tricuspid annulus and tethering of the tricuspid leaflets in an enlarged right ventricle.^10^, ^27^, ^28^ The right ventricle enlargement and RV dysfunction in severe AS patients is assigned to the chronicity and severity of pressure overload as a consequence of left‐side valve disease, AS, MR and pulmonary artery hypertension, and volume overload from fluid retention or the preexisting TR. TAVR can reduce LV hypertrophy but the degree of diffuse interstitial myocardial fibrosis is not changed. Diffuse interstitial myocardial fibrosis results in diastolic dysfunction and LV end‐diastolic over‐pressure, a possible cause of post‐capillary pulmonary hypertension.^8^, ^16^, ^29^ A meta‐analysis by Tang et al^30^ demonstrates pulmonary artery hypertension is associated with increased mortality. Patients with post‐capillary pulmonary hypertension, in comparison with pre‐capillary or combined pulmonary hypertension, are more susceptible to permanent myocardial damage and irreversible pulmonary vascular remodeling.^30^, ^31^ Therefore, severe symptomatic AS patients with baseline TR and RV dysfunction may not well relieve in post‐capillary pulmonary hypertension after TAVR. What is more, the improved stroke volume after TAVR increases systemic venous return, which could accelerate the dilation and failure of the right heart when combined with pulmonary hypertension.^11^, ^32^, ^33^

According to the ASE guideline,^22^ TAPSE, FAC, S′, and RIMP (also called Tei Index) are used to assess the RV systolic function. However, in the presence of TR, two‐dimensional echocardiography used to assess RV function is affected because TR may mislead the judgment of tricuspid leaflets. Cardiac magnetic resonance (CMR) may be a better choice yet not suitable for all patients because of the contrast.^16^ In subgroup meta‐analysis of different assessment method, we could conclude that FAC and S′ are not associated with outcome significantly, which is possibly related to TR. Even though TAPSE assumes that the displacement of a single segment represents the function of a complex 3D structure, it is a positive predictor similar to results reported by others.^16^, ^34^ Fortunately, RIMP is not affected by TR and is a more powerful predictor. Among echocardiographic measurements, the myocardial performance index is calculated as the ratio of the isovolumic contraction and relaxation time to the ejection time. Thus, right index of myocardial performance (RIMP) is a useful parameter reflecting myocardial relaxation and contraction. In some studies,^35^, ^36^ RIMP is a more long‐term powerful prognostic parameter in moderate or advanced heart failure. In conclusion, among the preoperative RV dysfunction patients, especially those with an abnormal value of RIMP which may indicate prior right heart failure, TAVR does not improve the pulmonary artery hypertension well, at the same time, it increases systemic venous return and right heart load, resulting in increased all‐cause mortality.^16^, ^35^, ^36^

Some studies^8^, ^9^ demonstrate that RV size is also evaluated as one of the independent predictors of outcomes after TAVR. First, RV dilation reflects chronic and severe pressure and volume overload,^6^ thus can be considered to be an advanced performance of RV dysfunction. Significant RV dilation could even be regarded as an advanced stage of right heart failure. Second, since the right ventricle shares the same septum with the left ventricle, RV dilation possibly causes left ventricular volume change.^9^ Third, patients with RV dilation are more likely to have AF, low LVEF, and chronic lung disease. This is in accordance with our meta‐analysis, RV dilation is an independent predictor of outcomes after TAVR.^9^

### 4.1 Study limitation

Our study has several limitations: (a) this was a meta‐analysis of nine studies, and there may be some bias; (b) our meta‐analysis only assessed the impact of preoperative TR and RV dysfunction on outcomes after TAVR, without taking consideration of evolution of TR and RV function post‐TAVR; (c) there was a moderate‐to‐high heterogeneity in the study for RV size, meta‐regression could be performed if there were more relevant studies.^37^ Taking into consideration the small group of studies and moderate‐to‐high heterogeneity, the result should be explained cautiously however, the following sensitivity analysis showed exactly the same result. Despite these limitations, our analysis provided valuable insights into the effect of right heart function on outcomes after TAVR.

## 5 CONCLUSION

Both baseline moderate or severe TR and RV dysfunction worsen prognosis after TAVR. RV dilation is additionally related to increased all‐cause mortality after TAVR. Careful assessment of right heart function should be done for clinical decision by the heart team before the TAVR procedure. More scientific studies and attention on right heart function is warranted in TAVR era.

## CONFLICTS OF INTEREST

The authors declare no potential conflict of interests.

## Supporting information


**Figure S1** Hazard ratio of RV size on all‐cause mortality after TAVR after removing one study

**Figure S2** All‐cause mortality outcomes after TAVR. Forest plot showing the individual and pooled analysis for (A) clinical factor adjusted hazard ratio of TR on all‐cause mortality. B, clinical factors and echocardiographic factors adjusted hazard ratio of TR on all‐cause mortality

**Table S1** Description of RV dysfunction and RV size in included studies
