# Supplementary figures and images for: Impact of tricuspid regurgitation and right ventricular dysfunction on outcomes after transcatheter aortic valve replacement: A systematic review and meta‐analysis
Source: Clin Cardiol. 2018 Dec 22;42(1):206–12. doi: 10.1002/clc.23126 (PMC6436507; doi:10.1002/clc.23126)

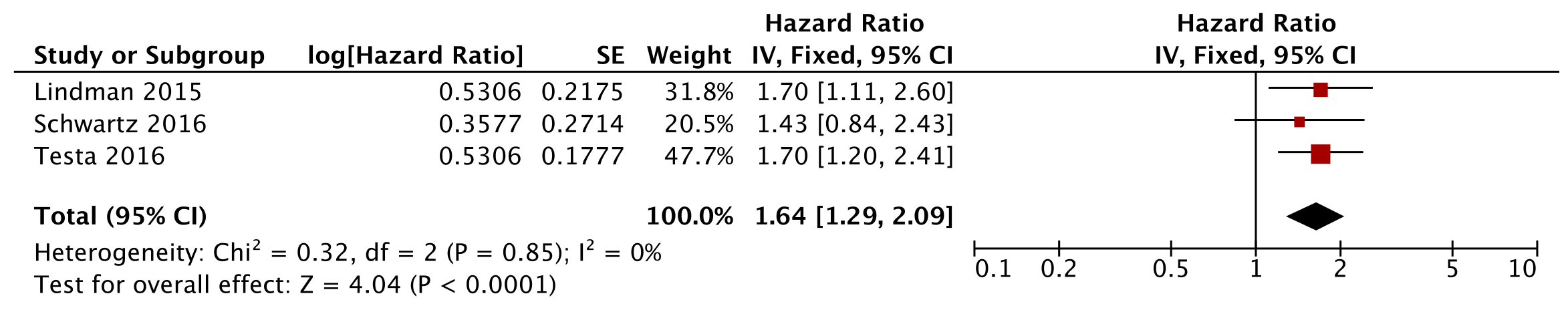

Supplement: Supplementary file 1 — Figure S1 Hazard ratio of RV size on all‐cause mortality after TAVR after removing one study [file CLC-42-206-s003.tiff]

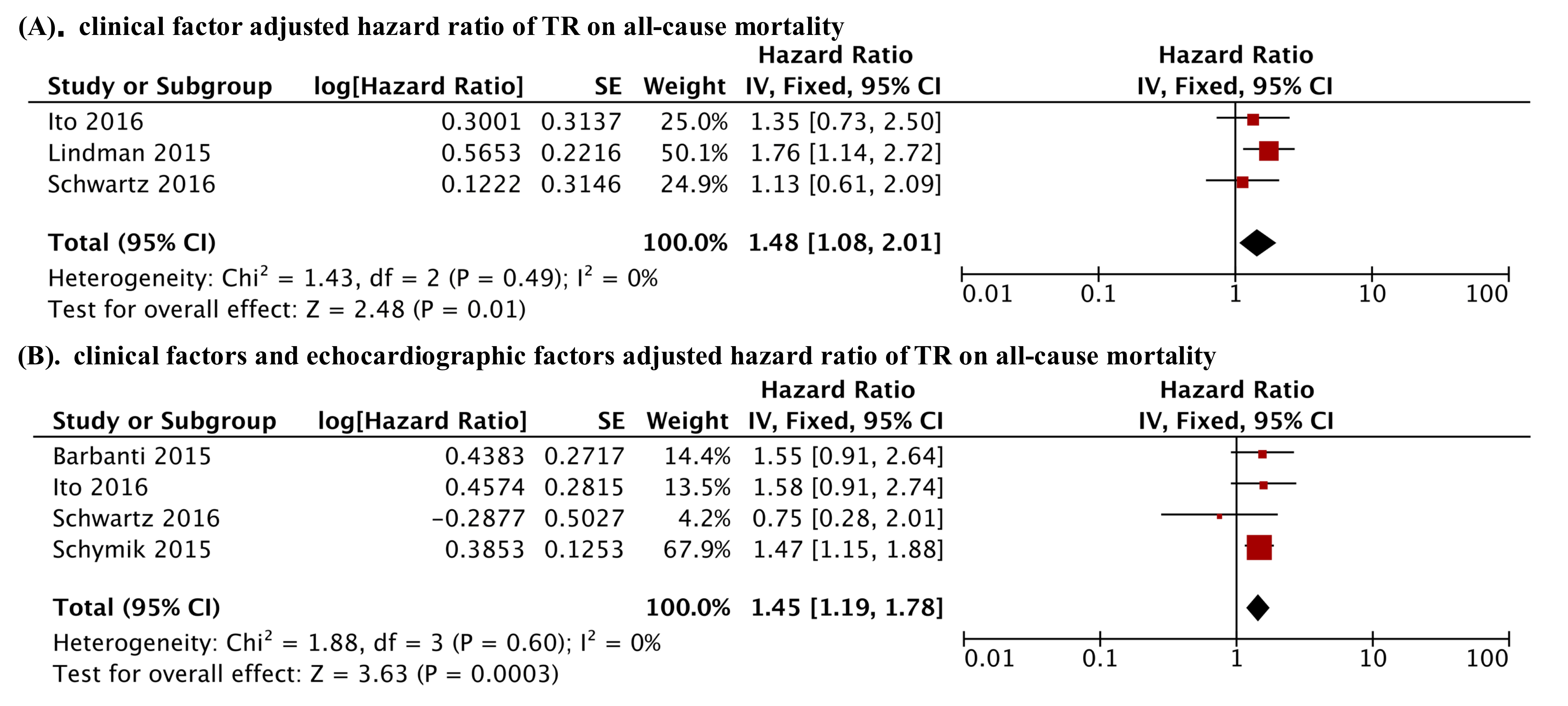

Supplement: Supplementary file 2 — Figure S2 All‐cause mortality outcomes after TAVR. Forest plot showing the individual and pooled analysis for (A) clinical factor adjusted hazard ratio of TR on all‐cause mortality. B, clinical factors and echocardiographic factors adjusted hazard ratio of TR on all‐cause mortality [file CLC-42-206-s001.tiff]
