# Supplementary material for: Impact of tricuspid regurgitation and right ventricular dysfunction on outcomes after transcatheter aortic valve replacement: A systematic review and meta‐analysis
Source: Clin Cardiol. 2018 Dec 22;42(1):206–12. doi: 10.1002/clc.23126 (PMC6436507; doi:10.1002/clc.23126)
Supplement: Supplementary file 3 — Table S1 Description of RV dysfunction and RV size in included studies [file CLC-42-206-s002.docx]

Supplementary Table 1 Description of RV dysfunction and RV size in included studies

| Study | Description of RV dysfunction | Description of RV size |
| --- | --- | --- |
| Lindman | Systolic and diastolic area and RV FAC | The basal and mid RV diameter and base-to-apex length in the 4 chamber view |
| Schwartz | TAPSE, RV end-systolic area, FAC, and myocardial performance index (Tei index) | End-diastolic and end-systolic areas, annular diameter |
| Ito | Systolic excursion velocity (S′) of the tricuspid lateral annulus, TAPSE, FAC, RIMP | RV longitudinal diameter, RV mid cavity diameter and RV basal diameter measured from a 4-chamber view |
| Lindsay | RV ejection fraction (cut-off is 50%, assessed by CMR) | NA |
| Poliacikova | TAPSE < 15mm or RVPSV < 9cm/s | NA |
| Testa | TAPSE < 16mm | RV longitudinal diameter, RV mid cavity diameter and RV basal diameter measured from a 4-chamber view |
| Griese | TAPSE < 18mm | NA |

*CMR*, Cardiovascular Magnetic Resonance; *FAC*, fractional area change; *RIMP*, right ventricular index of myocardial performance; *RV*, right ventricular; *RVPSV*, right ventricular peak systolic velocity; *TAPSE*, tricuspid annular plane systolic excursion.
